# Which concentrations are optimal for *in vitro* testing?

**DOI:** 10.17179/excli2020-2761

**Published:** 2020-08-18

**Authors:** Wiebke Albrecht

**Affiliations:** 1 Leibniz Research Centre for Working Environment and Human Factors, Ardeystr. 67, 44139 Dortmund, Germany

## ⁯⁯⁯⁯⁯

***Dear Editor,***

Currently, many research activities in the field of *in vitro* models focus at predicting human organ toxicity, e.g. of the liver (Gomez-Lechon et al., 2014[4]; Grinberg et al., 2014[6], 2018[5]; Frey et al., 2014[3]), kidney (Li et al., 2013[12], 2014[11]; Sjögren et al., 2018[15]; Sjögren and Hornberg, 2019[16]), heart (Nemade et al., 2018[13]; Chaudhari et al., 2018[2]) or developmental toxicity (Krug et al., 2013[9]; Waldmann et al., 2014[17]; Shinde et al., 2017[14]). All studies face a similar challenge, which is the choice of concentrations for *in vitro* testing (Leist et al., 2017[10]). This question has recently been discussed in an editorial of the Archives of Toxicology, where the authors pointed out that *in vitro *tests are usually performed at a concentration range around and above the plasma peak concentrations (C_max_) of a drug in humans (Hengstler et al., 2020[8]). A typical strategy is to test relatively high concentrations, often 20- or even 200-fold higher than the human plasma C_max_. The choice of high concentrations was justified by the observation that usually higher concentrations are required in the culture medium to induce cell damage compared to the C_max_ that is known to cause adverse effects *in vivo*. We made a similar observation in a recent study using human hepatocytes (Albrecht et al., 2019[1]; Gu et al., 2018[7]). The factor by which *in vitro* concentrations have to be higher than the corresponding *in vivo* plasma concentration in order to cause similar biological effects in the target cells is the *in vitro-in vivo* scaling factor. 

However, it should be considered that it is not yet clear if all compounds require identical *in vitro-in vivo* scaling factors. It cannot be excluded that e.g. compounds, whose toxicity depends on specific metabolic pathways may require higher scaling factors than compounds that do not require bio-activation. Moreover, scaling may also depend on the mechanism of toxicity (Hengstler et al., 2020[8]). To gain more insight into the requirements of optimal scaling it is not helpful to test only one or two concentrations, e.g. 20- and/or 200-fold the C_max_ and on this basis decide if a compound is positive or negative in the *in vitro* assay. Rather a concentration-dependent test with not too high dilution factors, such as 2- or at most 3.16-fold is helpful to be able to precisely determine the concentration when toxic effects occur, expressed e.g. as EC_10_ or EC_50_. This is a precondition to be able to elucidate if groups of compounds categorized e.g. by metabolic activation or mechanism of action require different scaling factors. A limitation to be overcome in the future is that too few studies established high quality, reproducible concentration response relationships for sufficiently high numbers of test compounds that allow a systematic comparison to the human *in vivo *situation. 

## Conflict of interest

The author declares no conflict of interest.
